# Role of Toll-Like Receptors and Th Responses in Viral Myocarditis

**DOI:** 10.3389/fimmu.2022.843891

**Published:** 2022-04-19

**Authors:** Shi-Yue Zheng, Jian-Zeng Dong

**Affiliations:** ^1^ Department of Cardiology, Beijing Anzhen Hospital, Capital Medical University, Beijing, China; ^2^ Department of Cardiology, The First Affiliated Hospital of Zhengzhou University, Zhengzhou, China

**Keywords:** viral myocarditis, TLRs, Th cells, regulatory T cell, immune response

## Abstract

Myocarditis is the common cause of sudden cardiac death, dilated cardiomyopathy (DCM) and heart failure (HF) in young adults. The most common type of myocarditis is viral myocarditis (VMC). Toll-like receptors (TLRs) are vital to identify pathogens *in vivo*. TLRs promote the differentiation of naive CD4^+^T cells to T helper (Th) cells, activate the immune response, and participate in the pathogenesis of autoimmune and allergic diseases. Although the pathogenesis of VMC is unclear, autoimmune responses have been confirmed to play a significant role; hence, it could be inferred that VMC is closely related to TLRs and Th responses. Some drugs have been found to improve the prognosis of VMC by regulating the immune response through activated TLRs. In this review, we discuss the role of TLRs and Th responses in VMC.

## Introduction

Myocarditis is a myocardial inflammation resulted from infectious, idiopathic, or autoimmune causes, of which the most popular is viral infection brought by enterovirus, Epstein-Barr (EB) virus, or human herpesvirus 6. Besides, myocarditis is the primary cause of dilated cardiomyopathy (DCM) and gradually becomes a cause of sudden cardiovascular death among young people (< 40-year-old) (1). Most patients with myocarditis can recover fully; however, some (up to 20%) develop chronic myocarditis, eventually resulting in DCM and heart failure (HF) (2). Myocarditis is diagnosed by combining clinical presentation, biomarkers, electrocardiogram (ECG), echocardiography, cardiac magnetic resonance imaging (CMRI), and endocardial biopsy (EMB). Tissue taken from EMB should be combined with the results of histology, immunohistochemistry and viral polymerase chain reaction (PCR) for the diagnosis of myocarditis (3). The EMB histology of myocarditis showed a value of leukocytes>14/mm^2^ with T lymphocytes>7/mm^2^, while immunohistochemistry showed an increase in the number of CD3^+^T cells or CD68^+^macrophages or CD163^+^M2 macrophages and virus genome could be detected by viral PCR (4, 5). EMB is a non-targeted operation, with low sensitivity as its main shortcoming, which may occur false-negative results when VMC is multifocal, focal, or localized (4). Therefore, the sensitivity of EMB in fulminant myocarditis with extensive inflammatory infiltration is increased, while that in focal myocarditis is relatively low, which may lead to false-negative results. In addition, although the virus can replicate in the myocardium, it does not cause enough myocardial inflammation, and EMB detection of the virus genome may also show false-negative results. The diagnostic criteria of CMRI for myocarditis are based on the ‘Lake-Louise’ criteria (6, 7). Therefore, in order to improve the sensitivity of EMB in diagnosis of myocarditis, we can determine the sampling site by combining CMRI and obtain myocardial tissue from three different sites. Chronic myocarditis also has persistent myocardial inflammation, which is the intermediate stage between acute myocarditis and chronic inflammatory cardiomyopathy. However, there is no detectable inflammation due to myocardial fibrosis in patients with chronic inflammatory cardiomyopathy, which renders diagnosis and treatment rather challenging (8).

Although the pathogenesis of myocarditis is yet unclear, the role of immune response in its process is under intensive focus. Under physiological conditions, a small number of immune cells are detected in the myocardium. After the onset of infections or autoimmune disorders, numerous immune cells and cytokines gather in the myocardial tissue to initiate inflammatory reactions. This process requires the initiation and maintenance of congenital and adaptive immune systems. Toll-like receptors (TLRs) recognize endogenous and exogenous ligands and are expressed on various cells, such as macrophages, neutrophils, dendritic cells (DCs), mast cells, and natural killer (NK) cells (9). They transmit signals to downstream pathways to stimulate innate and adaptive immunity after identifying the ligands involved in the pathogenesis of various autoimmune diseases, such as systemic lupus erythematosus (SLE), rheumatoid arthritis (RA), multiple sclerosis (MS), experimental autoimmune encephalitis (EAE), and experimental autoimmune myocarditis (EAM) (10). TLR1-TLR10 mRNA can be detected in normal peripheral blood T cells, but only TLR2-TLR5 and TLR9 expression can be detected at the protein level. TLR1, TLR2, and TLR7 are overexpressed on mRNA level in patients with myocarditis (9, 11). In addition, the inflammatory factors produced after TLRs activation, including interferon-gamma (IFN- γ), interleukin (IL)-6, and tumor growth factor-beta (TGF- β), can also stimulate native CD4+T cells to differentiate into T helper (Th) cells and participate in immune response to aggravate myocarditis. This study reviewed the role of TLRs and Th responses in viral myocarditis (VMC).

## VMC

VMC is the most common myocarditis caused by various viruses, including enterovirus, adenovirus, influenza virus, EB virus, and parvovirus; the most common is Coxsackievirus B3 (CVB3) that belongs to enterovirus (12). CVB3 may be cleared by innate immune response or stimulate the immune system to produce autoantibodies against the infection. The condition can be cured or progressed to DCM and HF (13). According to the clinical characteristics, VMC can be classified into fulminant, acute, subacute, or chronic myocarditis and localized or diffuse inflammatory infiltration can be observed in myocardial pathology. Fulminant myocarditis is rare and characterized by diffuse inflammatory infiltration in myocardial tissue, which has multiple active lesions and can be completely relieved, die, or progress to chronic myocarditis. Acute or chronic myocarditis progresses latently, resulting in DCM or HF (14). The pathological progression of VMC has three phases at the cellular and tissue level: the acute phase caused by viral entry and replication, the subacute phase characterized by inflammatory cell infiltration, and the chronic phase characterized by cardiac remodeling (15). VMC can be diagnosed by combining biomarkers, ECG, echocardiography, CMRI, and EMB (16). With the continuous update and development of technology, viruses in patients with VMC can be detected by polymerase chain technology, but EMB is still the gold standard for the diagnosis of myocarditis (3, 17, 18). VMC can be divided into eosinophilic, lymphocytic, giant cell, and granulomatous myocarditis based on the histological types observed by EMB. The most common type is lymphocytic myocarditis, wherein the main infiltration is by CD4^+^T and CD8^+^T lymphocytes, accompanied by CD68^+^ macrophages and few B lymphocytes (19, 20). However, only 38% of patients with VMC present viral genomes in their EMB samples (21). Hence, a close correlation is established between virus infection and immune response in the pathogenic process of VMC, while many studies have confirmed that the core of innate immunity and adaptive immunity is related to TLRs (22).

## TLRs

TLRs were first discovered as *Drosophila* gene and related to the human immune response (23). They are vital receptors on cells to recognize pathogens and belong to the pattern recognition receptor family (PRRs). They can detect pathogen-associated molecular patterns (PAMPs), such as unmethylated cytosine-phosphate-guanine DNA (CpGor TLR3, TLR7, TLR8, and TLR9 transport) and PRAT4A (responsible for TLR1, TLR2, TLR4, and TLR7 transport). These TLRs can only be functional after transport to the internal lysosome (24). Moreover, TLRs can also be heterodimerized, which expands the range of cognitive ligands. Different TLRs correspond to various endogenous ligands that are TLR4 and TLR2 agonists. The abnormal activation of TLRs may lead to unrestricted inflammatory response (25).

## Characteristics of TLRs

Hitherto, 11 TLRs have been found in humans (**Table 1**) (26). TLRs, such as TLR1, TLR2, TLR4, TLR6, and TLR10, are expressed on the cell surface and can recognize microbial membrane components, such as p-DNA), single-stranded RNA (ssRNA), double-stranded RNA (dsRNA), lipopolysaccharide (LPS), and flagellin and initiate immune response (27). In addition to the above exogenous ligands, TLRs can also recognize endogenous ligands, including high mobility group box 1 (HMGB1), heat shock proteins (HSP), human cardiac myosin (HCM) peptides S2-16, and HCM S2-28 (28, 29). Moreover, TLRs activate various types of cells and are highly expressed in most immune cells, chondrocytes, endothelial cells, and fibroblasts (30). All TLRs consist of an amino-terminal domain and a carboxyl-terminal Toll/interleukin-1 receptor (TIR) domain. The TIR domain interacts with the junction proteins, including myeloid differentiation factor 88 (MyD88), MyD88 adaptor-like (Mal, also known as TIR domain-containing adapter protein (TIRAP)), TIR domain-containing adaptor inducing IFN-β (TRIF), TRIF-related adaptor molecule (TRAM), and sterile a- and armadillo motif-containing protein (SARM), stimulating nuclear factor-kappa B (NF-κB) and the production of various proinflammatory cytokines, thereby initiating an immune response (31). Some TLRs, including TLR1, TLR2, TLR4, TLR5, TLR6, and TLR10, are expressed on the cell surface, while others, including TLR3, TLR7, TLR8, and TLR9, are expressed on the intracellular vesicles (32). Intracellular TLRs exist in the endoplasmic reticulum (ER) and are transported by ER resident proteins to the plasma membrane or lysosomes after stimulation: UNC93B (responsible f proteins, lipids, and participate in the recognition of virus proteins (33).

**Table 1:** Characteristics of TLRs.

TLRs	Localization	Ligands	Signaling pathways	Cytokines
TLR1 (with TLR2)	Cell surface	Triacylated lipopeptides	MyD88/TIRAP-NF-κB	TNF-α, IL-8
TLR2	Cell surface	HSP, HMGB1, HCM	MyD88/TIRAP-NF-κB	TNF-α, IL-8, IFN-γ, IL-12, IL-6
TLR3	Intracellular vesicle	Virus dsRNA	TRIF-IRF-3/NF-κB/AP-1	IFN-α/β
TLR4	Cell surface	HSP, Gp96, HMGB1	Mal/MyD88-NF-κB and TRIF/TRAM-type 1 IFN	IL-1β, TGF-β, TNF-α, IL-12 p40, IFN-α/β
TLR5	Cell surface	Flagellin	MyD88-NF-κB and p38 MAPK	IL-8, TNF-α
TLR6 (with TLR2)	Cell surface	Diacylated lipopeptides	MyD88/TIRAP-NF-κB	IFN-γ, IL-12, IL-6
TLR7	Intracellular vesicle	Virus ssRNA	MyD88-IRF-7	TNF-α, IL-12 p40
TLR8	Intracellular vesicle	Virus ssRNA, HCM	MyD88-NF-κB and MyD88-IRF-1/4/7	IL-1β, TNF-α, IL-6, IL-12, IFN-α/β
TLR9	Intracellular vesicle	Unmethylated CpG-DNA	MyD88	NF-κB, IL-1β, IL-18, IFN-α/β
TLR10	Cell surface	Lipopeptides	(–)	Inhibit IL-6, IL-10, TNF α, IL-1β

TLR2 forms heterodimers with TLR1 or TLR6 and recognizes different TLR ligands, resulting in different functions: dimers combined with TLR1 can recognize triacylated lipopeptides from bacteria while diacylated lipopeptides with TLR6 (34). Both TLR1/2 and TLR2/6 signaling pathways activate downstream inflammatory cytokines, tumor necrosis factor-alpha (TNF-α), (IL-8, IFN-γ, IL-12, and IL-6, through MyD88/Mal-NF-κB signaling pathway (35–37). When recognizing dsRNA, TLR3 transmits signals through TRIF and activates the transcription factor interferon regulatory factor 3 (IRF-3), NF-κB, and AP-1 (the complex of transcription factor 2 and jun), inducing the production of IFN-α/β, cytokines, or chemokines and the maturation of DCs (38, 39). TLR4 is the first molecule identified among TLRs and is mainly expressed in myeloid immune cells and in some non-immune such as endothelial cells (40). TLR4 can recognize heat shock protein, oxidized phospholipid, heparan sulfate, fibrinogen, fibronectin, tendon protein-C, and hyaluronic acid (41). Similar to other TLRs, TLR4 interacts with the intracellular TIR domain responsible for signal transduction (42). It mainly recruits Mal and MyD88 to activate NF-κB and utilizes TRIF and TRAM to activate type 1 IFN to produce proinflammatory factors, such as IL-1β, TGF-β, TNF-α, and IL-12 p40, to eliminate bacteria (43, 44). TLR5 activates the innate immune response against flagella by inducing a MyD88-dependent signaling pathway that stimulates the proinflammatory transcription factor NF-κB in epithelial cells, monocytes, and DCs (45). IL-8 and TNF-α can also be induced by the p38 mitogen-activated protein kinase (MAPK) signaling pathway in response to flagellin infection (46). TLR7 and TLR8 are homologous and located on the X chromosome. Both recognize virus ssRNA and are expressed in various immune cells (47). TLR7 is mainly expressed in plasma-like DCs and B cells, while TLR8 is mainly expressed in monocytes or macrophages, myeloid DCs, and neutrophils (48). Inflammatory factors, such as TNF-α and IL-12 p40 are activated through the MyD88-IRF-7 pathway after TLR7 activation, promoting the innate immune cells to perceive endosomal ssRNA, detecting RNA virus infection (49, 50). However, the overexpression or overactivation of TLR7 promotes the reduction of B cells producing IL-10 in an IFN-γ signal transduction-dependent manner and suppresses the immune response (51). TLR8 induces NF-κB through MyD88 signal transduction and promotes the expression of inflammatory factors, such as IL-1β, TNF-α, IL-6, and IL-12 after recognizing ssRNA. It also induces the production of IFN-α/β through IRAK4, IRAK1, and IRF-7 in response to viral infection (52). TLR9 was first cloned and identified as the receptor of unmethylated CpG-DNA in 2000. It induces the expression of IFN-α/β and proinflammatory cytokines (NF-κB, IL-1β, and IL-18) and activates the immune response only by recruiting MyD88 (53). TLR8 modulates the function of TLR7 on DCs, and TLR9 restrains the response of TLR7 on B cells. TLR7 crosstalk with TLR8 and TLR9 and play a critical role in the immune response of the body (54). Intriguingly, TLR10 is known as an orphan receptor because it lacks classical downstream signaling pathway. It is also an inhibitory receptor, homologous to TLR1 and TLR6, and hence, can form heterodimers with TLR2 and inhibit the production of proinflammatory cytokines, such as IL-6, IL-10, TNF-α, and IL-1β (46). It also inhibits monocyte differentiation, reduces the ability of DCs to stimulate T cells, and suppresses the immune response (55). The human *TLR11* gene has no function due to the presence of a stop codon (25).

## TLRs and Th Responses

**Figure 1:**
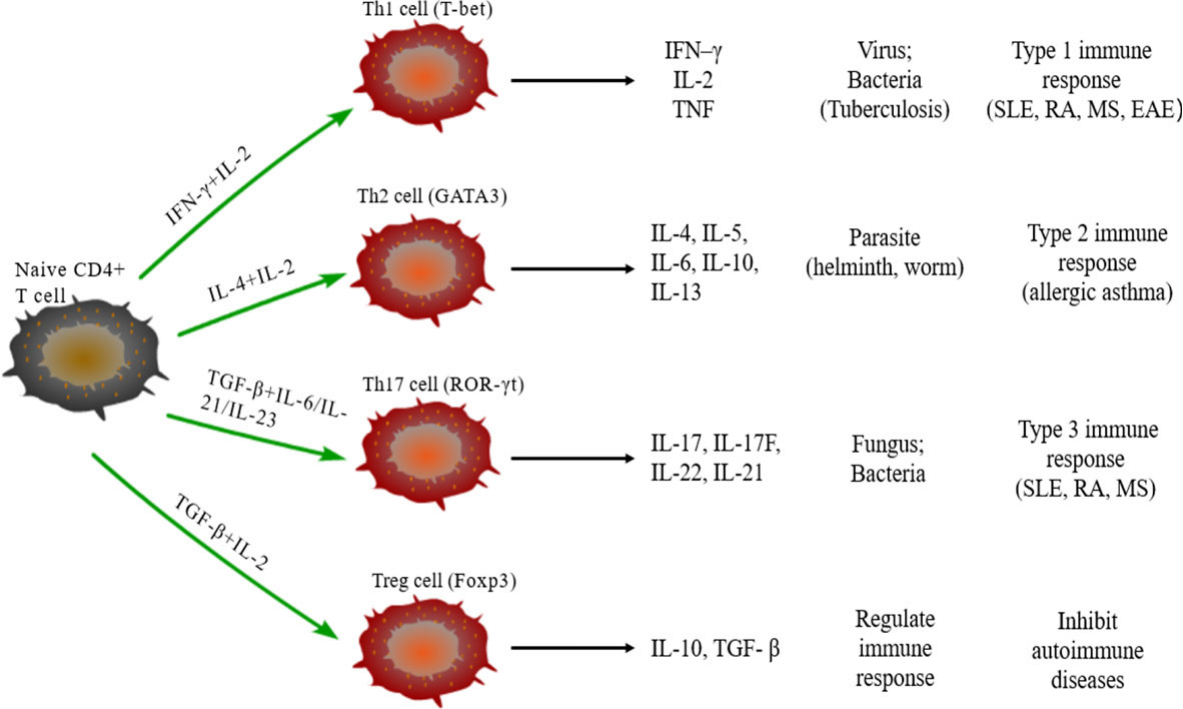
Differentiation of naïve CD4^+^T cells. Naive CD4^+^T cells can differentiate into Th1 cells under the stimulus of IFN-γ and IL-2, secrete IFN-γ, IL-2, and TNF, and participate in type 1 immune response. Under the stimulus of IL-2 and IL-4, naive CD4^+^T cells differentiate into Th2 cells that can secrete IL-4/5/6/10/13 and participate in type 2 immune response. The differentiation of Th17 cells need TGF-β, IL-6, IL-21, and IL-23, and participate in type 3 immune response through secreting IL-17/17F/21/22; TGF-β and IL-2 are required for naïve CD4^+^T cells to differentiate into Treg cells that secrete IL-10 and TGF-β regulate the immune response.

Naive CD4^+^T cells can differentiate into different subtypes of CD4^+^Th cells under the stimulus of cytokines. CD4^+^Th cells direct the immune response and play key roles in pathogenic infection, chronic inflammation, autoimmune diseases, and cancer. Some studies have found a variety of CD4^+^Th cells, such as Th1, Th2, Th17, and regulatory T (Treg) cells (**Figure 1**) (56). Naive CD4^+^T cells differentiate into Th1 cells post-stimulation of IL-2 and IFN–γ and expression of transcription factors T-bet and secrete IFN-γ, IL-2 and TNF. Th1 cells enhance cell-mediated inflammation and participate in type 1 immune response to intracellular pathogens, such as mycobacteria and viruses (57). Th2 cells are activated by IL-4 and IL-2 and are defined by the expression of transcription factor GATA3, subsequently secreting IL-4, IL-5, IL-6, IL-10, and IL-13. The Th2 cells also participate in type 2 immune response against large extracellular pathogens, such as worms, and play a role in the production of antibodies and allergic reactions (58). Th17 cells, stimulated by TGF-β, IL-6, IL-21, and IL-23 and the expression of transcription factor ROR-γt, produce IL-17, IL-17F, IL-22, and IL-21, which leads to tissue inflammation and promotes participation in type 3 immune response of extracellular pathogens, including bacteria and fungi. Different from other Th cells, Tregs differentiate under the stimulation of IL-10 and TGF-β and the expression of transcription factor Foxp3 to produce anti-inflammatory cytokines, IL-10 and TGF-β (59). Moreover, Tregs inhibit autoimmune diseases and regulate immune response to maintain immune cell homeostasis. Type 1 and 3 immune responses mediate autoimmune diseases, such as SLE, RA, and MES, while type 2 immune responses can lead to allergic diseases, such as asthma (60). Cytokines crosslink each other to maintain Th cells balance. IFN-γ and IL-4 antagonize each other at different levels, and hence the development of Th1 and Th2 cells is mutually exclusive (61). Th17 cells can promote the development of autoimmunity, while Treg cells inhibit autoimmunity; thus, the imbalance of Th17/Treg cells in the body is considered the leading mechanism underlying autoimmune diseases (62).

**Figure 2:**
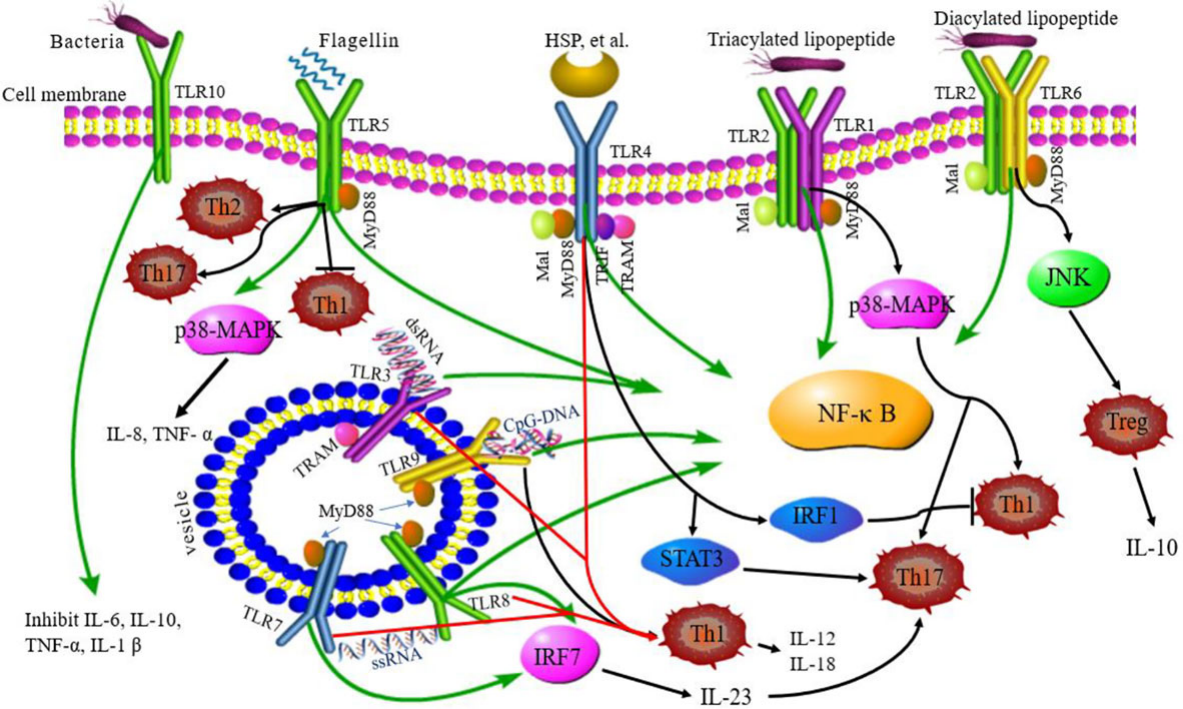
TLRs signaling pathway and related Th responses. TLR1, TLR2, TLR4, TLR5, TLR6, and TLR10 are expressed on the cell surface, while TLR3, TLR7, TLR8, and TLR9 are expressed on intracellular vesicles. TLRs can recognize different ligands and recruit adapter proteins, MyD88, Mal, TRIF, or TRAM, activating the downstream signaling pathway. TLR10 is an orphan receptor and lacks a classical downstream signaling pathway. Some TLRs promote the differentiation of Th cells. TLR2/6 promotes Treg cell differentiation through the JNK pathway. TLR2/1 promotes Th1 and Th17 cell differentiation through p38 MAPK pathway. TLR4 eliminates the Th1 response through IRF1 and promotes Th17 cell differentiation by STAT3 pathway. TLR5 can stimulate the body to induce Th2 response, inhibit Th1 response, and promote DC differentiation into Th17 cells. TLR8 induces the expression of IL-12B and IL-23A, promotes the differentiation of IL-23-dependent Th17 cells. TLR9 promotes the differentiation of Th1 cells. Green lines: TLR signaling pathways; black lines: TLRs related to Th cell differentiation; red lines: co-stimulation of TLRs induced to Th1 cell.

The activation of TLRs has been shown to bridge innate immunity and acquired immunity. In addition to expression in antigen-presenting cells (DCs and macrophages), TLRs are also expressed in T cells playing a costimulatory role in T cell activation and inducing Th cell differentiation (**Figure 2**) (63). TLR2 promotes the differentiation of Th17 cells and immune response by disrupting the balance of Th17/Treg cells (64). TLR2/6 ligand is a bacterial lipopeptide that can induce DC tolerance and promote the differentiation of IL-10-producing Tregs through the c-Jun N-terminal kinase (JNK) pathway both *in vivo* and *in vitro*. On the other hand, the activation of TLR2/1 promotes the DCs to produce a high level of IL-12 p40 and a low level of IL-10 through p38 MAPK signaling pathway, thereby triggering the differentiation of Th1 or Th 17 cells (65). TLR4 eliminates Th1 response through IRF1 and IFN-α/β receptor-dependent mechanisms. The lack of TLR4 promotes Th1 cell differentiation by enhancing STAT1 pathway, inhibits Th17 cell differentiation by inhibiting STAT3 pathway, and interferes with immune response (66, 67). Bacterial LPS also aggravates allergic inflammation through the production of Th2 cytokines and participates in the immune response of the body post-TLR4 activation (68). Soluble bacterial flagellin stimulates the body to induce Th2 response through TLR5 and inhibits Th1 response to bacterial infection (69). TLR5 promotes DCs in the intestinal tract to differentiate into Th17 cells and respond to pathogen invasion (70). TLR8 induces the expression of IL-12B and IL-23A, promotes the differentiation of IL-23-dependent Th17 cells, and produces immune responses after activating human neutrophils (71). The co-stimulation of TLR7/8 ligands and TLR4 or TLR3 ligands produce IL-12p70 that is the key cytokine to induce Th1 immune response (72). Therefore, ligand co-stimulation is crucial to induce Th1 response. TLR9 is essential in the production of proinflammatory cytokines and other inflammatory responses and to initiate Th1 response and B cell proliferation (73). The interaction between CpG-DNA and TLR9 rapidly activates DCs through the Toll/IL-1 receptor signaling pathway, promoting the differentiation of Th1 cells and the production of cytokines (IL-12 and IL-18) (74). TLR9 ligands also bind to Th cells to promote the proliferation of cells and upregulate the cytokines (75). TLRs-induced immune response is involved in various diseases (9). Presently, several TLR agonists are being tested as adjuvants in the treatment of autoimmune diseases by balancing the immune response.

## Role of Th Responses in VMC

Th responses play an important role in the pathogenesis of VMC, but different Th responses have different effects on VMC, which may have opposite effects. Besides, the dominant Th responses are different in different stages of VMC.

## Th1/Th2 Responses

**Figure 3:**
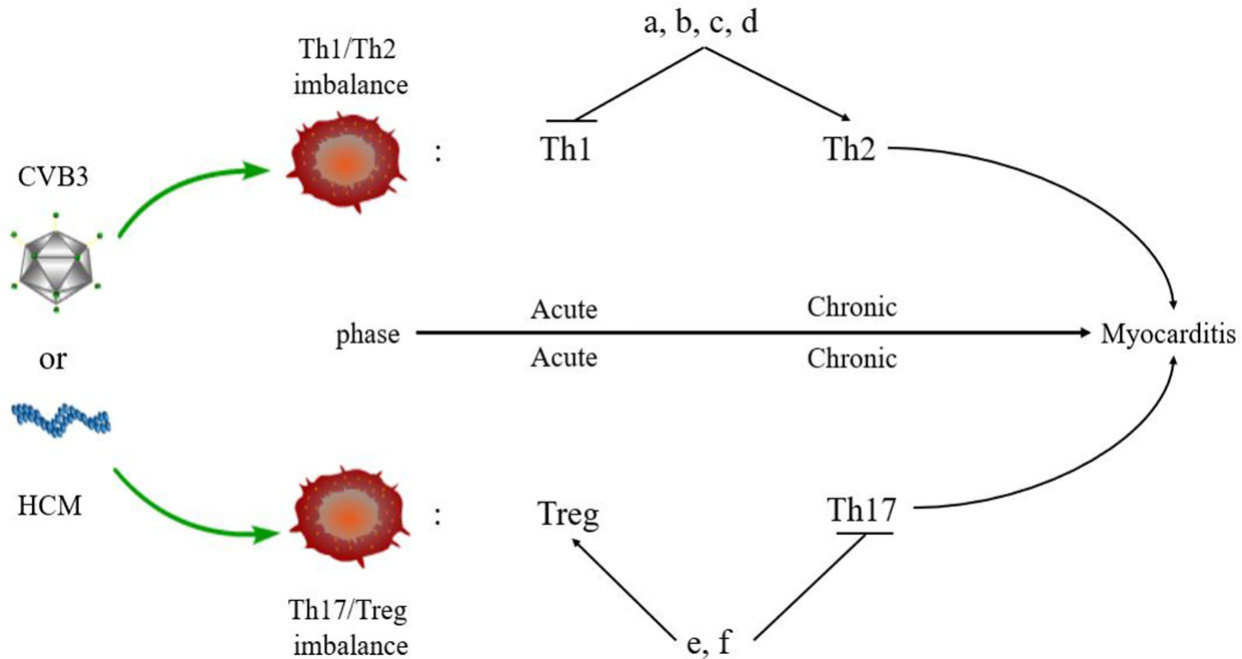
Myocarditis and Th responses. An imbalance of Th cells is observed in the pathogenesis of myocarditis. Th1 and Treg cells are predominant in the acute phase of myocarditis, while Th2 and Th17 cells dominate the chronic phase of myocarditis. Some drugs regulate the balance of Th cells in myocarditis. **(A)** Atorvastatin; **(B)** Tanshinone IIA; **(C)** Apigenin; **(D)** Cyclooxygenase-2 inhibitors; **(E)**. Valproic acid; **(F)** Fenofibrate.

The imbalance of Th1/Th2 cells can be observed in the process of VMC (**Figure 3**). Fuse et al. (76) observed the changes in Th1/Th2 ratio of peripheral blood lymphocytes in a patient with acute VMC. In the acute inflammatory phase (day 6), Th1 cells were dominant, while in the recovery phase (days 13 and 20), the proportion of Th2 cells increased. The induction of VMC was related to the dominance of Th1 cells, while the recovery was related to the increased proportion of Th2 cells. However, Th2 immune response induces ventricular remodeling that promotes myocarditis to develop into DCM and HF in the pathogenesis of VMC, while Th1 response alleviates VMC by inhibiting Th2 response and virus replication, but increases acute myocardial inflammation (77). Therefore, when Th2 response begins to be active, the inflammation of VMC decreases, and if Th2 response persists, it will promote myocardial fibrosis and ventricular remodeling. The study also demonstrated that Suramin (a growth factor blocker) inhibits myocardial inflammation in myocarditis by regulating the environment of Th1/Th2 cytokines (78). Therefore, elucidating the Th1/Th2 response might help to understand the activity of VMC. Based on these results, several drugs, such as atorvastatin, tanshinone IIA, apigenin, and cyclooxygenase-2 inhibitors, have been shown to have protective effects on rat model of myocarditis by regulating Th1/Th2 balance (79–82). However, the above drugs can promote Th2 response, which may aggravate the progression of VMC to DMC or HF. Therefore, it is necessary to clarify the therapeutic effect of drugs in the stage from myocarditis to DCM or HF.

## Th17/Treg Responses

Th17 cells secreted IL-17, promoting myocardial fibrosis after myocarditis through protein kinase C β/extracellular signal-regulated kinases 1 and 2/NF-κB pathway, which is an indispensable link in the process of DCM (83). In addition, Tregs can protect mice from CVB3-induced myocarditis progression to cardiomyopathy (84). CVB3 infection stimulates the differentiation of Th17 cells and promotes the secretion of IL-17 by inhibiting the expression of Nucleoporin 98 and aggravating VMC (85). In the acute phase of VMC, Th17 cells stimulate B cells to produce autoantibodies and participate in humoral immune response. The Th2 cells participate in humoral immune response at the late stage of VMC, which is consistent with the above conclusion (86). In the pathogenesis of VMC, the imbalance of Th17/Treg cells plays a critical role in the immune mechanism. MicroRNA-155 (miR-155) is a key regulator of the immune system and promotes the development of myocarditis *via* differentiation of Th17 cells leading to the imbalance of Th17/Treg cells. The inhibition of miR-155 relieves myocardial injury and the disease (87). Other drugs, such as valproic acid and fenofibrate have also been found to inhibit inflammation, reduce CVB3-induced VMC, and improve prognosis by directly inhibiting the differentiation of Th17 cells (88). Thus, VMC can be treated by promoting the differentiation of Treg cells and regulating the balance of Th17/Treg cells (89). In addition, estrogen inhibits the differentiation of Th17 cells that are mainly induced in males with CVB3 infection but less in females. Thus, Th17 cells show gender bias in myocarditis: the incidence of myocarditis has a male-to-female ratio of 2:1 (90). This indirectly indicates that Th17/Treg cell balance plays a key role in the epidemiological characteristics of myocarditis.

## Role of TLRs in VMC

**Figure 4:**
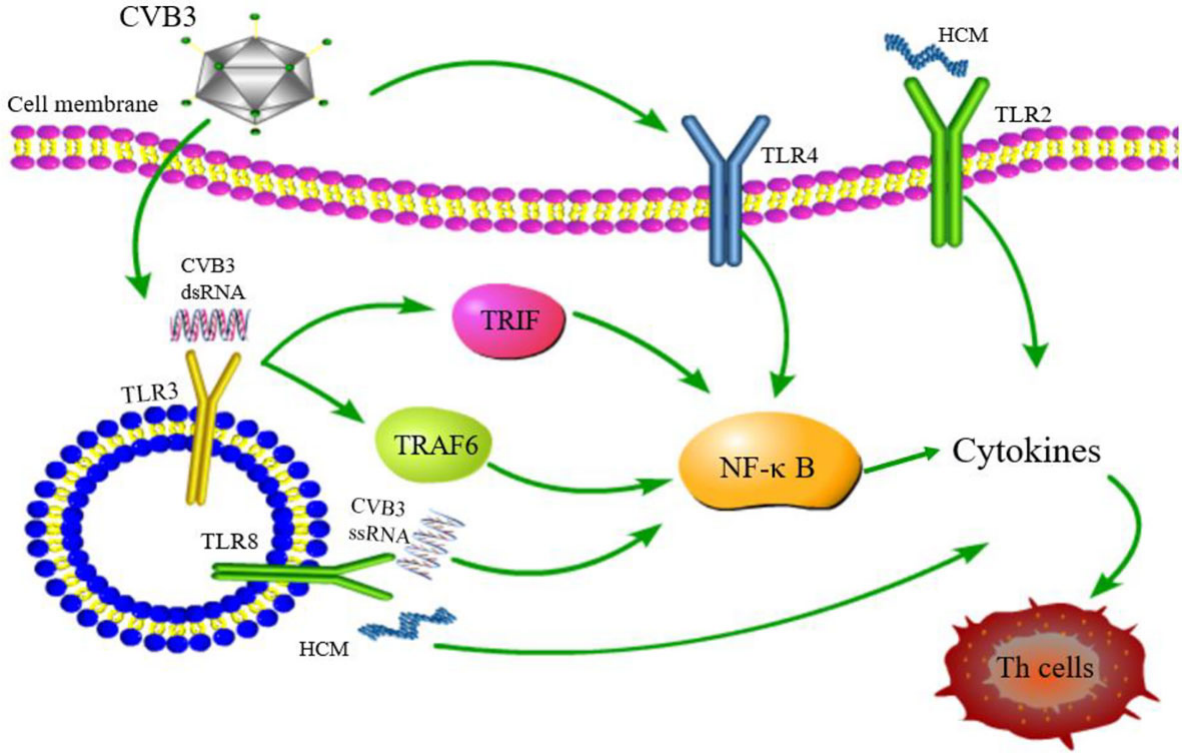
TLR signaling pathway related to CVB3 infection. TLR4/NF-κB is activated after CVB3 infection; TLR3 recognizes CVB3 dsRNA and activates TRIF and TRAF6 to transmit signals to NF-κB; TLR8 recognizes CVB3 ssRNA and activates NF-κB signaling pathway. TLR2 and TLR8 recognizes HCM and promotes the differentiation of Th cells. Although other TLRs may be related to the pathogenesis of myocarditis, whether other TLRs can be activated and its downstream pathway after CVB3 infection has not been reported.

As key members of PRRs, TLRs participate in the upstream signaling pathway that activates innate immune cells and T cells, resulting in the production of proinflammatory cytokines and the activation of T cells. TLRs are considered to be the main factors in the development of autoimmunity, participating in and promoting the occurrence of autoimmune inflammatory diseases (91). The above observations indicate gender differences in the incidence of VMC. Roberts et al. (92) demonstrated that high expression of TLR2 in early infected female mice exerted a protective effect, while that of TLR4 in male mice was lethal. This differential expression between genders resulted in disease resistance in female mice and susceptibility in male mice (**Figure 4**). Hence, TLRs are deemed to play a critical role in gender difference with respect to myocarditis and understanding the underlying mechanisms would illuminate the epidemiological characteristic of myocarditis (93). TLR3 recognizes dsRNA intermediates produced during CVB3 replication and activates TRIF and TRAF6 to transmit signals to NF-κB (94). TLR3-TRIF signaling pathway helps the host to defend against CVB3 infection. The mechanism might be ascribed to the induction of type II IFN expression, rather than IFN-α/β, and TLR3-TRAF6-III IFN signaling pathway also has antiviral effects (95, 96). The lack of TLR3 increases virus replication and aggravates myocardial inflammation. It also worsens cardiac function and increases the susceptibility to CVB3 (97). The genetic variation of TLR3 affects the host’s susceptibility facing VMC by inhibiting the signal transduction of NF-κB (21). These results proved that TLR3 has a protective effect on the myocardium in the process of virus infection. In addition, neutrophils also interact with and recognize CVB3 through TLR8, activating NF-κB and its downstream factors, resulting in VMC development (98). It also upregulates the expression of TLR4, promotes the expression of NF-κB, and induces myocarditis (99). Based on these results, astragalus polysaccharides have been shown to protect TLR4-induced myocardial injury and inflammation by inhibiting the CVB3-related signaling pathway (100), which provides a potential target to treat myocarditis. TLR7 preferentially promotes the differentiation of Th17 cells and the expression of inflammatory factors, such as IL-17 after CVB3 infection, while TLR8 promotes the production of Th1 cytokines and IFN-α/β response, which are involved in the pathogenesis of myocarditis (101). The potent autoantigen HCM is released from damaged heart during viral infection. HCM peptides S2-16 and S2-28, as an endogenous ligands, can bind to TLR2 and TLR8, and promote the release of pro-inflammatory factors such as IL-8, IL-6, IL-23 and TGF-β, which mainly induce the differentiation of Th17 cells and contribute to DCM or HF (29, 102). Although TLR9 can recognize various DNA viruses unlike the indirect way of recognizing RNA viruses, TLR9-MyD88 signaling pathway mediates myocardial injury in acute phase rather than chronic phase CVB3-induced myocarditis (103). Nonetheless, the mechanisms of other TLRs in myocarditis are yet to be clarified.

## Conclusions

TLRs and Th responses play a critical role in the pathogenesis of VMC and have become the focus of current research. TLRs are a new class of innate immune receptors that mediate CD4+T cell differentiation, induce Th1 and Th2 immune responses, and participate in VMC pathogenesis. Except that CVB3 can directly bind to TLRs to promote Th responses, the release of HCM from damaged heart can also promote DCM or HF through TLRs and Th responses after viral infection. Blocking or activating a single TLR or regulating TLR signaling pathway may affect innate immunity, host resistance, and VMC pathogenesis, indicating that specific TLRs agonists or antagonists comprise new immunotherapy for VMC. In addition, some anti-inflammatory drugs have been found to reduce myocardial injury and improve VMC by interfering with TLR signaling pathways and Th immune responses. However, the role of other TLRs and Th responses in VMC has not yet been reported. Therefore, clarifying the role of TLRs and Th responses in VMC can provide novel ideas for the treatment of VMC.

## Author Contributions

All authors contributed to the article and approved the submitted version.

## Conflict of Interest

The authors declare that the research was conducted in the absence of any commercial or financial relationships that could be construed as a potential conflict of interest.

## Publisher’s Note

All claims expressed in this article are solely those of the authors and do not necessarily represent those of their affiliated organizations, or those of the publisher, the editors and the reviewers. Any product that may be evaluated in this article, or claim that may be made by its manufacturer, is not guaranteed or endorsed by the publisher.
